# Present Day Problems

**Published:** 1888-11-24

**Authors:** 


### THE DOCTOR'S PULPIT.

### PRESENT-DAY PROBLEMS.

### THE QUESTION OF POPULATION.

MR. W. E. GLADSTONE and other writers have recently drawn public attention to the rapid increase of population which has taken place in English-speaking countries during the last century. According to their showing, if the present rate of increase continues, as it probably will, we shall have in Britain alone in the time of our grandchildren seventy or eighty millions of people; and in the United States and Canada nearly ten times that number. In other words, the world bids fair to become in a hundred years an English-speaking world. The practical question that the man of business asks himself is, What are all these people going to eat, and where is their food to come from? The question will not interest some; for there are persons who, so long as they know that to-morrow morning's breakfast is in the cupboard when they go to bed at night, care for no more. But there are other men who cannot help caring. They see that not only will the pressure of population be severe in 1988, but that it is severe now. They are conscious that the problem presses for solution in London and elsewhere at the present moment. They think, too, that if it be clearly and wisely solved now, our solution may help those who come after.

Has medicine as a science and an art anything to do with this problem? Has physiology anything to do with it? Undoubtedly! On the health of the people depends the wealth of the people; on their physique their prosperity is balanced. Mark the stages of a horse's history. At first a promising colt, then a splendid hunter, then a sober hack, and finally a "bag of bones" in a four-wheeled cab. Let it be supposed that the horse has descendants. The progeny of his hunting days will inherit his most splendid qualities; they will be raised in plenty, and trained by the best skill of the time. But any progeny he may be followed by in his "bag-of-bones" stage will inherit his feeble and broken-down constitution, will be bred in his miserable surroundings, or killed, as the tramway companies kill "baby horses," because it does not pay to rear them.

This is a more or less faithful picture of what often happens, and now more than ever, to human beings. In the olden times most Britons were stalwart, country-bred men. Their descendants, bred like them in the country, followed the laws of heredity and the stimulus of a wholesome environment. They, too, became stalwart men, who could plough, or build, or fight, or ride the seas, or do anything else that destiny required of them. But as times have progressed the men of the country have become men of the town. The hunters have been gradually degraded to the "bag-of-bones" stage. Descendants have been born to them in this stage; they have been bred in the most miserable of environments; in their turn they too have become progenitors of other still smaller and feebler descendants. We are now surrounded by thousands of such inferior human stock, and the downward tendency is increasing on all hands. These are facts. The farmers of the State, be they politicians, parsons, doctors, lawyers, or men of business, are confronted with wretched human stock, bare pastures, and an atmosphere choked with almost every variety of pestilence.

Let no butterfly thinker call this mere rhetoric. It is a fact, as hard, bare, and evident as the sea-cliffs of Shetland or of Norway. The physiologist and the doctor, if they have any scientific brains at all, feel that whilst horses, and cattle, and sheep, and pigs, and foxes are committed to farmers, squires, and landowners, human creatures—surely the noblest of all live stock—are committed to them. They, if not altogether responsible, are at least more responsible than any other class for the improvement of the breed of men, for the development of splendid specimens, for the production of races which shall have bones, muscles, blood, and brains. Why have the peoples of India gone down before the Englishman? Because the Englishman has hitherto been, like his own racehorse, swifter, higher couraged, more enduring than those against whom he has been matched in the race. For this, and for no other reason.

But the Englishman is on the decline. And why is he on the decline? For one of two reasons: either because the population is too great for the means of subsistence, or because the means of subsistence are misused. In which of these two directions the truth may lie, there is contained the whole secret of national decadence or national advancement. What does medical science say on the subject? It says that the two imperative conditions of prosperity and progress for a race is that the race, as a whole, shall have the means of subsistence in abundance, and shall have free scope for increase, without undue mental and physical strain. That is both the law and the gospel of physiological and medical science on this subject. Artificial methods of restricting the growth of population are artificial methods of promoting national destruction.

It is certain that we have now reached a time when the mouths of the whole population, and those of individual families, have become too numerous for both the national and the family bread-loaf. The State feels the burden of its people, and most parents feel the burden of their children. A house full of boys and girls is not now looked upon with that easy and generous parental satisfaction which used to animate parents two or three generations ago. In addition to the needs of the moment, which are infinite in variety and urgent in intensity, there is the uncertain outlook for the future. What shall be done with the boys? What can be done with the girls? These questions press on the attention of father and mother, morning, noon, and night.

Now natural laws, as scientists call them, are apt to be very imperative and very unyielding. Physiology and disease take no account of difficulties. Whether there be food in the cupboard or not, the waiting stomach becomes hungry; whether the clothing be warm and sufficient or thin and scanty, the wintry winds will blow and the frosts will chill the marrow. There are other natural appetites which demand reasonable satisfaction, and the demand cannot be neglected without injury and danger. Whatever be the mountains that stand in the way, the physical and mental needs of man are peremptory, and not to be denied. We repeat, that physiology and medicine both declare two of the essential conditions of the prosperity and progress of a race to be the means of subsistence in abundance, and free scope for natural increase without undue physical and mental strain.

*(To be continued.)*

